# Epidemiology of pertussis in Denmark, 1995 to 2013

**DOI:** 10.2807/1560-7917.ES.2016.21.36.30334

**Published:** 2016-09-08

**Authors:** Tine Dalby, Peter Henrik Andersen, Steen Hoffmann

**Affiliations:** 1 Statens Serum Institut, Microbiology and Infection Control, Copenhagen, Denmark; 2 Statens Serum Institut, Department of Infectious Disease Epidemiology, Copenhagen, Denmark

**Keywords:** pertussis, vaccine-preventable diseases, surveillance, respiratory infections, laboratory surveillance

## Abstract

We describe incidence and age distribution of laboratory-confirmed pertussis in Denmark from 1995 to 2013. Notification has been mandatory since 2007. Since 1997, an acellular monocomponent vaccine has been used. The latest epidemic occurred in 2002 with an incidence of 36 per 100,000; since 1995, only six infant deaths have been recorded. The inter-epidemic incidence lies below 10 per 100,000. In 1995, the mean age of confirmed cases was 9.2 years (95% confidence interval (CI): 7.9–10.5; median: 5.1), this gradually increased to 23.9 years in 2013 (95% CI: 22.0–25.8; median: 15.7). In 1995, 14% of laboratory-confirmed cases were 20 years and older, 43% in 2013. In the study period, the highest incidence among children was among those younger than one year with incidences between 84 and 331 per 100,000 in inter-epidemic periods (mean: 161/100,000) and 435 for the epidemic in 2002. After introduction of a preschool booster in 2003, the highest incidence among children one year and older changed gradually from three to five-year-olds in 2003 to 12 to 14-year-olds in 2013. In 2013, PCR was the primary method used for laboratory-diagnosis of pertussis in Denmark, while serology was the method with the highest percentage of positive results.

## Introduction

Pertussis (whooping cough) is a highly contagious respiratory tract infection caused by the bacterium *Bordetella pertussis*. In the early 20th century before the introduction of vaccines, pertussis was the cause of extensive morbidity and numerous infant deaths. In Denmark during 1900 to 1959, 19–53% of all infants contracted pertussis, and up until the 1930s, ca 10% of cases had a fatal outcome [1]. With the introduction of vaccines in the mid-19th century the incidence of pertussis decreased markedly all over the world, but more than 50 years into the vaccine era, pertussis is still prevalent and causes substantial outbreaks, even in countries with a long history of vaccination. In 2008, pertussis was the cause of an estimated 195,000 deaths worldwide among children younger than five years, primarily in the developing world [2]. In Denmark during 1920 to 1929, 2,569 infants died from pertussis, but in the years 1995 to 2013, only six deaths from pertussis were recorded and all were infants younger than two months, i.e. too young to have started vaccination [3,4].

In countries where vaccines have almost eliminated infant deaths due to pertussis, the knowledge about pertussis in the general population has also declined. Thus, many falsely believe pertussis to be solely a childhood infection, and a common misperception is that pertussis vaccination will protect you for life. In reality, immunity after pertussis vaccination or even after an episode of pertussis is only short-lived, at an estimated four to 12 years or four to 20 years, respectively [5], or five to 10 years as a rule of thumb. Consequently, all age groups in the population can contract pertussis, and adult pertussis is not as rare as commonly thought. Several reports have shown that the key sources of pertussis infection for vulnerable non-vaccinated infants are parents or other adults in close contact (reviewed in [6]). Since pertussis in adults can present as just a prolonged cough without the characteristic whoop or the post-tussive vomiting [7-9], it is important to realise that pertussis is a disease of all age groups. With increased knowledge, novel vaccination strategies and improved diagnostic methods, the transmission of pertussis to non-vaccinated infants can hopefully be diminished. This paper summarises the epidemiology of laboratory-confirmed pertussis in Denmark during the period from 1995 to 2013 as well as the distribution of laboratory-methods used across the whole of Denmark in 2013 for the diagnosis of pertussis.

## Background on pertussis vaccination and diagnostics in Denmark

Vaccination against pertussis was introduced in Denmark in 1961 as a whole-cell (wP) vaccine [10]. In 1997, this vaccine was replaced by an acellular vaccine (aP) with pertussis toxoid (PTx) as the sole pertussis antigenic component (Table 1) and in 2003, a preschool booster at five years of age was introduced. The aP vaccines used in Denmark are unique compared with other aPs around the world in that (i) they contain high amounts of PTx at 40 µg for the infant series and 20 µg for the preschool booster, and (ii) the toxoid is prepared by hydrogen peroxide detoxification of the pertussis toxin, rather than the formaldehyde and/or glutaraldehyde inactivation used by many other manufacturers (reviewed in [11]). Detoxification by hydrogen peroxide has been shown to result in a lower degree of epitope impairment of the toxin compared with formaldehyde, and the generated immune response may therefore be more effective [12,13]. Moreover, the Danish aP vaccines elicit a stronger antibody response against pertussis toxin than a number of other aPs [11,14,15].

**Table 1:** Historic overview of pertussis vaccines and schedules used in Denmark

Change	Vaccines	Schedule
1961	**wP** ( + DT)	5, 6, 7 and 15 months
1969	wP	**5 and 9 weeks, 10 months**
1997	**aP primary series, 40µg PT** ***(DTaP-IPV)***	3, 5 and 12 months
2002	aP primary series, 40µg PT *(DTaP-IPV****/PRP-T)***	3, 5 and 12 months
2003	aP primary series, 40µg PT *(DTaP-IPV/PRP-T)*	3, 5 and 12 months
	**aP booster, 20µg PT** ***(TdaP)***	**5 years**
2004	aP primary series, 40µg PT *(DTaP-IPV/PRP-T)*	3, 5 and 12 months
	aP booster, 20µg PT *(TdaP-****IPV)***	5 years

aP: acellular pertussis vaccine containing pertussis toxoid (PT) as the sole pertussis antigenic component; wP: whole-cell pertussis vaccine.

The composition of the vaccines is marked in italics according to international nomenclature. Changes are marked in bold letters.

Laboratory diagnosis of pertussis in Denmark was initially done by culture of the bacterium, and this was only done at the clinical microbiological laboratory at Statens Serum Institut (SSI). In 1998, PCR was introduced at SSI [16] and from 2002, public regional clinical microbiology laboratories gradually began using either culture or PCR. Subsequently, the proportion of pertussis diagnostics in Denmark performed at SSI decreased gradually from 100% in 1995 to 98% in 2002, 64% in 2006 and to 32% in 2013. For regional clinical microbiology laboratories not performing pertussis diagnostics, samples were, and still are, sent either to SSI or to one of the other regional clinical microbiology laboratories. Thus, the diagnostics for pertussis covered the whole of Denmark in the whole period from 1995 to 2013. In 2013, seven clinical microbiology laboratories in Denmark including SSI were performing diagnostic tests for pertussis. All were employing PCR, and the only laboratory also maintaining the culture of *B. pertussis* was SSI. At SSI, culture is performed on Regan-Lowe agar, and for the whole study period, the PCR was performed as an initial IS481/IS1001 PCR followed by a confirmatory ptxP PCR. Details on the PCR methods used at the regional laboratories are not available. In 2010, serology was introduced at SSI as an in-house IgG anti-PT ELISA with a cut-off at 75 IU/mL. The test is only considered valid for individuals eight years and older in order to avoid interference from antibodies elicited by the preschool booster normally given at five years of age [17,18]. Positive results from serology-confirmed children are only included in the database if the vaccination registry proves that the latest vaccination happened more than two years previously and if the child is older than six months; such cases are very few (16 during the study period).

Culture of *B. pertussis* has a specificity of 100%. However, the sensitivity is low compared with PCR and serology [19-21]. This is mainly due to the difficulty in obtaining a nasopharyngeal sample of sufficient quality, the low chance of retrieving viable *B. pertussis* [22], the long incubation time necessary for culture and the fact that culture is only reliable in the first two weeks of symptoms [23]. PCR is useful in the first three weeks of symptoms and is also dependent on a correctly obtained nasopharyngeal sample [23,24].

Serology is useful for diagnosis of patients having had symptoms for more than two weeks [23,25] and serology is therefore particularly efficient in diagnosing pertussis among adults, who can experience mild symptoms and therefore may be more likely to seek medical attention at a later stage of the disease, after some weeks with symptoms [7,26,27].

Initially, since only the laboratory at SSI performed diagnostics of pertussis, data on all laboratory-confirmed cases were registered at SSI. When regional clinical microbiology laboratories gradually began using PCR or culture for pertussis starting in 2002, they all voluntarily submitted their data on confirmed pertussis cases to SSI for surveillance purposes. This voluntary reporting was ongoing at full coverage until 2007 when it became mandatory to report all laboratory-confirmed cases of pertussis to SSI. The national database of laboratory-confirmed pertussis at SSI therefore covers the whole of the country since 1995, i.e. from before the mandatory reporting was initiated. Recently, a national database (The Danish Microbiology DataBase (MiBa)) has also been established which automatically receives electronic real-time copies of all test reports, whether positive or negative, from all Danish departments of clinical microbiology, making it possible to investigate the use of laboratory methods across the country [28,29]. In addition, cases of confirmed pertussis among children younger than two years need to be notified on a paper form. The national surveillance of pertussis in Denmark covers only laboratory-confirmed cases. Cases based on the clinical picture alone or based on an epidemiological link are not registered.

Deaths from pertussis were registered as part of a routine used in the period from 1995 to 2013 that systematically requested information from the notifying clinician on possible sequelae, including death, for all notified cases in children younger than two years.

During the whole period from 1995 to 2013, the national vaccination uptake remained at a high level, averaging 89% for the third primary vaccination for the birth cohorts 2003 to 2013. However, the method of calculation changed from using an administrative method between 1995 and 2005 to using a register-based approach (retrospectively) since 2006. In the period from 1995 to 2005, uptake of all three infant doses of the combination vaccine containing the acellular pertussis component, DTP3/DTaP, was in the range of 90–99% at 12 to 23 months of age. For the birth cohorts 2006 to 2013, DTaP uptake was in the range of 85–91%, with an increasing trend [30]. Regarding the five-year booster vaccination, coverage for the birth cohorts 2000 to 2008 was in the range of 81–87%, also with an increasing trend [31].

## Methods

Data from the Danish national database on laboratory-confirmed pertussis was analysed for the period from 1995 to 2013. Except for a few cases registered before the reporting of cases became mandatory in 2007 (68 cases in the period from 2002 to 2004 without date of birth, age or date of diagnosis, 20 cases in 2006 with age but without date of birth or date of diagnosis, 120 cases in the period from 2004 to 2006 with age and date of diagnosis but without date of birth), all entries include the Danish personal identification number (CPR number), date of birth, date of sampling for the test that confirmed the diagnosis and name of the laboratory that performed the diagnostic test. Information on diagnostic method was unavailable for entries before 2010.

Incidence was calculated based on annual population data from StatBankDenmark [32].

Data from the clinical microbiology laboratories in Denmark (through MiBa) [28] was analysed for samples submitted for pertussis diagnostics in the period from 1 January to 31 December 2013. In some instances, two simultaneous samples are sent from the same patient, and we therefore filtered the data so that samples taken on the same day and with the same result were treated as a single sample in the analysis.

We also analysed data from the national Danish database on notified cases of pertussis among children under the age of two years for the period from 1995 to 2013. This database also contains information on the patients’ vaccination status.

## Results

### Annual incidence

**Figure 1:**
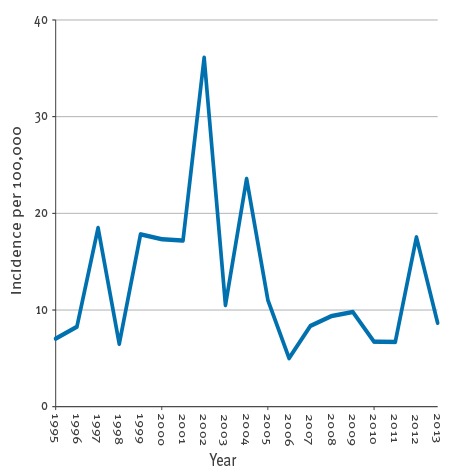
Incidence of laboratory-confirmed pertussis per 100,000 population, Denmark, 1995–2013 (n = 13,269)

**Figure 2:**
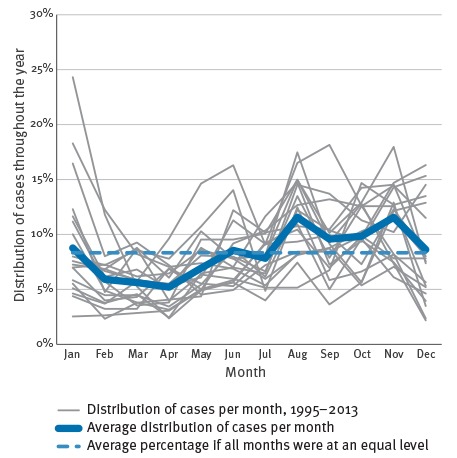
Seasonality of laboratory-confirmed pertussis, Denmark, 1995–2013 (n = 13,269)

Since 1995, the annual total number of laboratory-confirmed cases of pertussis in Denmark has ranged from 272 to 1,272, with an epidemic peak in 2002 at 1,938 cases. With a population ranging from 5.2 million in 1995 to 5.6 million in 2013 , this corresponds to annual incidences between 5 and 36 per 100,000. Apart from a peak in 2012 with 978 cases corresponding to an incidence of 18 per 100,000, the level has been stable since 2005 at a mean value of 451 cases annually, an incidence of 8 per 100,000 (Figure 1). At present, in early 2016, it has thus been 14 years since an epidemic of pertussis has occurred in Denmark. It is evident that pertussis in Denmark is not as seasonal as many other respiratory tract infections. When looking at the combined data for the whole period, there was however a tendency towards a lower occurrence in the months of February to April and a higher occurrence in the months of August to November (Figure 2).

### Age distribution

**Figure 3:**
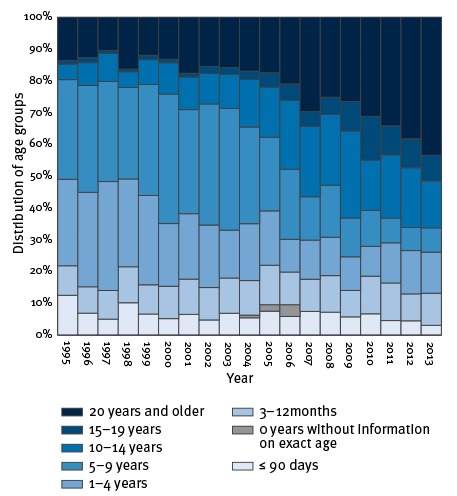
Age-distribution of laboratory-confirmed cases of pertussis, Denmark, 1995–2013 (n = 13,269)

**Figure 4:**
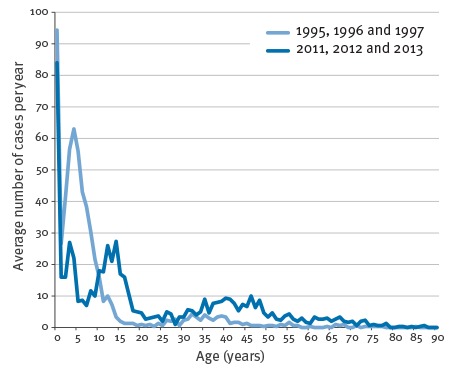
Average number of laboratory-confirmed pertussis for two periods by patient age, Denmark, 1995–97 (n = 1,776) and 2011–13 (n = 1,836)

The age distribution of cases shows that more and more adult cases are found (Figure 3 and Table 2). In 1995, 80% of all cases were found among children younger than 10 years but in 2013, this figure had decreased to 34%. Similarly, in 1995, cases among adults 20 years and older accounted for 14% of all cases but this figure increased to 43% in 2013. The median age of laboratory-confirmed pertussis gradually increased from 5.1 years in 1995 (interquartile range (IQR): 1.5–8.7) to 15.7 years in 2013 (IQR: 4.8–41.5). Moreover, due to the introduction of the preschool booster in 2003 the largest age group among older children diagnosed with pertussis changed from the 3–5-year-olds between 1995 and 1997 to the 12–14-year-olds between 2011 and 2013 (Figure 4). This age-specific peak shifted gradually after the booster was introduced (data not shown here, but described previously [11]). We chose the first three and the last three years in the study period for Figure 4 because the total number of cases for the two periods were comparable.

**Table 2:** Number and (incidence) of laboratory-confirmed cases of pertussis, Denmark, 1995–2013 (n = 13,269)

Age	1995	1996	1997	1998	1999	2000	2001	2002	2003	2004	2005	2006	2007	2008	2009	2010	2011	2012	2013
≤ **90 d^a^**	46	30	49	35	63	48	60	91	38	68	45	16	34	37	31	25	17	44	15
**0 years total**	80 (115)	66 (94)	137 (202)	74 (109)	150 (227)	142 (214)	162 (241)	285 (435)	99 (154)	215 (330)	131 (202)	54 (84)	80 (123)	96 (149)	76 (116)	69 (109)	61 (96)	127 (214)	64 (110)
**1–4 years**	100 (38)	129 (47)	334 (120)	95 (34)	266 (96)	182 (66)	189 (70)	375 (139)	83 (31)	224 (84)	102 (39)	28 (11)	56 (22)	62 (24)	57 (22)	35 (13)	47 (18)	133 (51)	63 (25)
**5–9 years**	115 (39)	146 (48)	307 (98)	99 (30)	331 (99)	376 (110)	301 (86)	726 (207)	211 (60)	381 (109)	138 (40)	60 (18)	62 (18)	84 (25)	66 (20)	42 (13)	29 (9)	71 (22)	37 (11)
**10–14 years**	18 (7)	31 (11)	86 (31)	17 (6)	74 (26)	91 (30)	94 (30)	182 (57)	59 (18)	187 (55)	94 (27)	59 (17)	101 (29)	114 (32)	148 (42)	59 (17)	74 (22)	184 (54)	72 (21)
**15–19 years**	4 (1)	7 (2)	9 (3)	3 (1)	13 (5)	10 (4)	11 (4)	42 (15)	12 (4)	33 (11)	27 (9)	14 (4)	21 (7)	28 (8)	50 (15)	51 (15)	34 (10)	89 (25)	39 (11)
**20–29 years**	8 (1)	12 (2)	18 (2)	14 (2)	19 (3)	24 (3)	20 (3)	46 (7)	17 (3)	32 (5)	10 (2)	11 (2)	30 (5)	26 (4)	22 (3)	25 (4)	20 (3)	54 (8)	25 (4)
**30–39 years**	25 (3)	23 (3)	46 (6)	25 (3)	49 (6)	51 (6)	85 (10)	129 (16)	32 (4)	80 (10)	37 (5)	18 (2)	33 (4)	40 (5)	32 (4)	24 (3)	32 (4)	99 (14)	52 (7)
**40–49 years**	11 (1)	12 (2)	15 (2)	8 (1)	22 (3)	18 (2)	25 (3)	61 (8)	18 (2)	49 (6)	27 (3)	11 (1)	42 (5)	29 (4)	55 (7)	34 (4)	39 (5)	115 (14)	71 (9)
**≥ 50 years**	6 (0.4)	8 (0.5)	23 (1)	9 (1)	23 (1)	29 (2)	32 (2)	59 (3)	20 (1)	51 (3)	30 (2)	17 (1)	30 (2)	34 (2)	34 (2)	33 (2)	36 (2)	106 (5)	63 (3)
**Unknown**	0	0	0	0	0	0	0	33	15	20	0	0	0	0	0	0	0	0	0
**Total**	367 (7)	434 (8)	975 (18)	344 (6)	947 (18)	923 (17)	919 (17)	1,938 (36)	566 (11)	1,272 (24)	596 (11)	272 (5)	455 (8)	513 (9)	540 (10)	372 (7)	372 (7)	978 (18)	486 (9)

Incidences are shown in brackets.

^a^ Incidence could not be calculated for infants ≤  90 days since populations statistics are not available for age categories shorter than one year.

### Infant pertussis and vaccinations

For the whole study period 1995 to 2013, the proportion of infant pertussis (in those younger than 12 months) among notified laboratory-confirmed cases younger than two years averaged 83%, ranging from 77% to 90%. The average proportion of unvaccinated cases among all cases younger than two years of age was 46%, ranging from 25% to 58%. Conversely, the average proportion of fully (three doses) vaccinated pertussis cases was 10%, ranging from 3% to 15%, indicating vaccine failures even within the first two years of life.

### Diagnostic methods

Laboratory data from MiBa on samples submitted for pertussis diagnostics in the whole of Denmark for the year 2013 (n = 4,569) showed that 79.6% of the samples were submitted for PCR (n = 3,639), 17.8% were submitted for serology (n = 811) and 2.6% were submitted for culture (n = 119). Seven laboratories performed diagnostics for pertussis. All seven used PCR, while only one laboratory (SSI) also performed culture and serology. When looking at samples found positive for pertussis (n = 520), 72% were found by PCR (n = 374), 28% were found by serology (n = 143) and 0.6% were found by culture (n = 3). The number of positive samples was higher than the reported number of cases, as two or more samples were often submitted from the same patient. The proportions of positive results for each of the three methods were 10% for PCR (374/3,639), 18% for serology (143/811) and 2.5% for culture (3/119). The proportion of positive results (confirmed pertussis) for PCR methods in the seven individual laboratories ranged from 6.5% to 13.7%. Sera submitted as part of a diagnostic serology package for atypical pneumonia at SSI were positive for pertussis in 11% (39 of 368) of the samples, while sera submitted solely for diagnosis of pertussis at SSI were positive in 23 of the samples (104/443).

As mentioned previously, serology is particularly efficient when diagnosing pertussis among adults and although the method is still new in Denmark, it has already had an impact. It is moreover obvious, that the method is increasingly useful with increasing age of the patient. In fact, in 2012 and 2013, 27% of all laboratory-confirmed cases in Denmark for the age group 8–19 years were confirmed by serology. For the age group 20–49 years, this figure was 38%. When looking only at cases 50 years and older, 49% of these were confirmed by serology (Table 3).

**Table 3:** Number of laboratory-confirmed pertussis cases with information on the diagnostic method used for confirmation of pertussis, Denmark, 2010–13 (n = 13,269)

Age group	Method	2010		2011		2012		2013	
		n	%	n	%	n	%	n	%
0 years	Culture	4	6	3	5	7	6	0	0
	PCR	64	93	58	95	120	94	64	100
	Serology	1	1	0	0	0	0	0	0
1–7 years	Culture	2	4	2	3	3	2	1	1
	PCR	46	94	56	92	166	95	73	91
	Serology	1	2	3	5	5	3	6	8
8–19 years	Culture	8	6	4	3	4	1	2	2
	PCR	118	86	94	76	216	71	96	73
	Serology	12	9	25	20	83	27	33	25
20–29 years	Culture	3	4	3	3	6	2	0	0
	PCR	51	61	66	73	152	57	98	66
	Serology	29	35	22	24	110	41	50	34
≥ 50 years	Culture	2	6	3	8	3	3	0	0
	PCR	20	61	18	50	54	51	30	48
	Serology	11	33	15	42	49	46	33	52
Total	Culture	19	5	15	4	23	2	3	1
	PCR	299	80	292	78	708	72	361	74
	Serology	54	15	65	17	247	25	122	25

Percentages show the contribution of each method for each year.

## Discussion

Many countries have seen a resurgence of pertussis in recent years [33], but this has not been seen in Denmark where the latest epidemic occurred in 2002. The peak in 2012 coincided with peaks in many other European countries [34] and the high incidence in Denmark was therefore most probably influenced by the high incidence in neighbouring countries.

Unfortunately, the incidence in Denmark is difficult to compare to other countries as there are substantial differences in awareness, diagnostic practices, notification systems, population densities, communities that refuse vaccination etc., as illustrated in a number of publications [35-37]. Surveillance data from some of Denmark’s neighbouring countries exemplify this; the incidences per 100,000 in the years 2010 to 2013 were in the ranges 2 to 3 in Sweden, 52 to 90 in Norway, 4 to 10 in Finland, 0.8 to 18 in England and 21 to 82 in the Netherlands [38-42]. Australia had incidences in the range of 54 to 173 per 100,000 in the same period [43]. All these countries should be comparable in terms of general health and vaccination coverage.

The proportion of confirmed pertussis found among adults in Denmark in relation to the total number of confirmed cases has gradually increased, and almost half of all the cases in 2013 were adults. This shift in age groups has been seen in many other countries around the world [33,44-47] and is thought to be due to several factors, primarily improved awareness and improved diagnostic methods [48]. Moreover, the introduction of a five-year booster vaccination in 2003 shifted the peaks of age-specific incidences among children when comparing the period 1995 to 1997 with the period 2011 to 2013. Cases among unvaccinated infants can be used as an indicator for the actual occurrence of pertussis since the attention on infants suspected of pertussis will presumably always be high. The number of pertussis cases among up to 90 days-old infants has decreased from a mean inter-epidemic level of 47 per year in the period 1995 to 2001 to 29 in the period 2007 to 2013 (27 cases when discounting the peak in 2012). This could indicate that the true level of pertussis in Denmark has declined since the 1990s.

When comparing data across the study period, we assume that a gradual increase should have been observed, reflecting improved laboratory methods after the introduction of PCR in 1998 and serology in 2010. PCR has in fact been found to be five times more sensitive than culture [49], and a part of the increase from 1998 to 2002 is probably due to the introduction of PCR. However, the levels after 2004 were comparable to the levels before 1999. The enhanced awareness on adolescent and adult pertussis should also have had an impact. However, this has not been the case, and only six infant deaths from pertussis were registered in Denmark between 1995 and 2013, emphasising that the disease burden of pertussis is indeed low. The baseline level between peaks even decreased from 947, 923 and 919 cases, respectively, in the three years 1999, 2000 and 2001 to 455, 513, 540, 372 and 372 cases, respectively, in the five years from 2007 to 2011. A possible explanation could be that the introduction of the aP vaccine in 1997 and the preschool booster in 2003 lead to improved immunity in the population compared with the wP era. This is, however, purely hypothetical.

Analysis of the methods used for pertussis diagnostics in Denmark in 2013 shows that PCR was the most frequently used method while serology had the highest percentage of positive results. However, as PCR samples are often analysed routinely for a larger panel of infections, the proportion of positive results for the different assays should be compared with caution. Culture was rarely used and only few of the samples were positive. The diminished use of culture poses a problem in the sense that the circulating *B. pertussis* strains are not monitored. Recovery of isolates from PCR samples could perhaps compensate for this [50].

In the coming years, we expect to see a further increase in the numbers and proportion of confirmed pertussis among Danish adults. This change is not expected to be due to an increase in the true incidence, but rather to a virtuous circle of increased use of serology that will increase awareness of adult pertussis, leading again to increased test activity.
